# Influences of the biofeedback content on robotic post-stroke gait rehabilitation: electromyographic vs joint torque biofeedback

**DOI:** 10.1186/s12984-019-0558-0

**Published:** 2019-07-23

**Authors:** Federica Tamburella, Juan C. Moreno, Diana Sofía Herrera Valenzuela, Iolanda Pisotta, Marco Iosa, Febo Cincotti, Donatella Mattia, José L. Pons, Marco Molinari

**Affiliations:** 1 Spinal Rehabilitation Laboratory - Neurological and Spinal Cord Injury Rehabilitation Department A, Santa Lucia Foundation IRCCS, Via Ardeatina 306 –, 00179 Rome, Italy; 2 Laboratory of Robotics Applied to Neurological Rehabilitation- NeuroRobot - Neurological and Spinal Cord Injury Rehabilitation Department A, Santa Lucia Foundation IRCCS, Via Ardeatina 306 –, 00179 Rome, Italy; 3 Spanish National Research Council, Cajal Institute, Neural Rehabilitation Group, Av. Doctor Arce, 37, 28002 Madrid, Spain; 4 Department of Biomedical Engineering, Universidad de los Andes, Bogotá, Colombia; 5 Laboratory for the Study of Mind and Action in Rehabilitation Technologies – Smart Lab, Santa Lucia Foundation IRCCS, Via Ardeatina 306, 00179 Rome, Italy; 6 Department of Computer, Control and Management Engineering, Sapienza University of Rome, Rome, Italy; 7 Neuroelectrical Imaging and BCI Lab, IRCCS S. Lucia Foundation, Via Ardeatina 306 -, 00179 Rome, Italy; 8 Legs & Walking AbilityLab, Shirley Ryan AbilityLab, Chicago, IL USA; 9 Department of Physical Medicine & Rehabilitation, Feinberg School of Medicine. Department of Biomedical Engineering & Department of Mechanical Engineering, McCormick School of Engineering. Northwestern University, Chicago, IL USA

**Keywords:** Stroke, Rehabilitation, Robot, Biomechanics, Electromyography, Biofeedback, Top-down approach

## Abstract

**Background:**

Add-on robot-mediated therapy has proven to be more effective than conventional therapy alone in post-stroke gait rehabilitation. Such robot-mediated interventions routinely use also visual biofeedback tools. A better understanding of biofeedback content effects when used for robotic locomotor training may improve the rehabilitation process and outcomes.

**Methods:**

This randomized cross-over pilot trial aimed to address the possible impact of different biofeedback contents on patients’ performance and experience during Lokomat training, by comparing a novel biofeedback based on online biological electromyographic information (EMGb) versus the commercial joint torque biofeedback (Rb) in sub-acute non ambulatory patients.

12 patients were randomized into two treatment groups, A and B, based on two different biofeedback training. For both groups, study protocol consisted of 12 Lokomat sessions, 6 for each biofeedback condition, 40 min each, 3 sessions per week of frequency. All patients performed Lokomat trainings as an add-on therapy to the conventional one that was the same for both groups and consisted of 40 min per day, 5 days per week. The primary outcome was the Modified Ashworth Spasticity Scale, and secondary outcomes included clinical, neurological, mechanical, and personal experience variables collected before and after each biofeedback training.

**Results:**

Lokomat training significantly improved gait/daily living activity independence and trunk control, nevertheless, different effects due to biofeedback content were remarked. EMGb was more effective to reduce spasticity and improve muscle force at the ankle, knee and hip joints. Robot data suggest that Rb induces more adaptation to robotic movements than EMGb. Furthermore, Rb was perceived less demanding than EMGb, even though patient motivation was higher for EMGb. Robot was perceived to be effective, easy to use, reliable and safe: acceptability was rated as very high by all patients.

**Conclusions:**

Specific effects can be related to biofeedback content: when muscular-based information is used, a more direct effect on lower limb spasticity and muscle activity is evidenced. In a similar manner, when biofeedback treatment is based on joint torque data, a higher patient compliance effect in terms of force exerted is achieved. Subjects who underwent EMGb seemed to be more motivated than those treated with Rb.

## Background

Stroke is the leading cause of acquired disability throughout the world, with increasing survival rates as medical care and treatment techniques improve [1]. Post-stroke disability often affects mobility, balance, and walking [2]. The majority of stroke survivors rank walking recovery among their top rehabilitation goals [3–5]. Furthermore, the ability to walk is one of the most important determining factors for returning home after stroke [4].

Recovery of walking mainly occurs within the first 11 weeks after a stroke [6]; indeed, further recovery after that time is rare [7]. Overall, between 30 and 40% of stroke survivors are not able to regain a functional gait after rehabilitation [4, 8]. These data have stimulated advances in many different innovative technological approaches to improve the gait rehabilitation efficacy.

Modern concepts favour task-specific repetitive rehabilitation approaches [9], with high intensity [10] and early multisensory stimulation [11]. These requirements are met by robot assisted gait training (RAGT) approaches. Recent studies on stroke patients have reported that when conventional therapy and RAGT are combined, compared to conventional therapy alone, gait recovery significantly improves [12] and patients are more likely to recover independent walking [13]. In particular, non-ambulatory patients in the sub-acute phase are the group most likely to benefit from this type of training [13].

This high interest in robotic therapy has attracted attention to human robot interactions in the rehabilitation framework, and a consensus is forming on the importance of top-down approaches in rehabilitation, particularly when dealing with robotic devices [14]. The critical aspects of top-down approaches are multifarious and include motivation, active participation [15], learning skills [16] and error-driven-learning [17], evidencing the key aspects of biofeedback information to guide and improve patient robot interactions.

Thus, biofeedback is, at present, the main approach to guide top-down control mechanisms, which represents a powerful tool to drive recovery. To this aim, the patient has to be aware of the differences between on-line performance and the desired performance [18]. In this scenario, many different error signals can be used, and at present, no indication exists for their specific effects on performances [18, 19]. Many biological parameters have been used to feed biofeedback information to patients in different stroke gait rehabilitation scenarios [20].

In general, in spite of the information content, biofeedback has been associated with improved outcomes in several gait pathologies [21–24]. Among diverse types of biofeedback, the most generally employed in gait rehabilitation paradigms have been electromyographic (EMG), kinematic as well as robot generated indexes [25], although no comparisons have been made among these approaches.

At present, many robotic devices for gait rehabilitation in stroke are commercially available [26]. Two main classes can be identified, those based on body weight support systems (BWSS) and over ground exoskeletons. Overall, BWSS are the most widely used in rehabilitation centres, with Lokomat, Gait Trainer and GEO systems being the most popular. The present study focuses on the biofeedback content effects during Lokomat gait training in stroke survivors. Commercially available Lokomat biofeedback tools are based either on navigational or robot-generated information. The latter approach focuses on the forces that assist patients to follow the predefined gait pattern due to force transducers built into the robot drives [25].

Generally effectiveness of Lokomat training is assessed with gait functional outcome measures. Specific data about spasticity effects of Lokomat training are rare, and mainly focused on spinal cord injury (SCI) patients and on ankle muscles. In this framework few studies addressed positive effects of Lokomat training on reducing spasticity and improving volitional control of the spastic ankle in persons with incomplete SCI [27], and on reducing the abnormal modulation of neuromuscular properties that arises as secondary effects after SCI [28, 29]. To our knowledge, as concern stroke population, a single study compared conventional rehabilitation versus Lokomat add-on training selecting spasticity as a secondary outcome, demonstrating no significant robotic gait training effects [30].

Furthermore, no studies have either analysed the use of an electromyographic -based biofeedback (EMGb) of hip, knee and ankle muscles during training with the Lokomat robot, or compared the impact of different biofeedback types on Lokomat robotic gait training. To this end, we designated a randomized controlled trial, because this type of study is the most rigorous and robust research method of determining whether a cause-effect relation exists between an intervention and an outcome [31]. In this pilot study we compared two different types of biofeedback: a robot generated joint torque biofeedback (Rb) versus a novel on-line EMGb. Thus, a randomized cross-over clinical trial using the Lokomat RAGT device, was conducted focusing on patients’ performances, personal experience and robot forces data in sub-acute non ambulatory patients. In particular the main outcome measure was considered the lower limb spasticity. Considering that in stroke population, spasticity may affect quality-of-life and can be highly detrimental to daily function [32], we also analysed patients’ personal experience related to training gait with the Lokomat system.

## Methods

### Patients enrolled

A randomized cross-over design was selected for this pilot study that aimed to compare EMGb versus Rb effects on patients’ performance, personal experience perception and robot measurements in non-ambulatory sub-acute stroke patients. The primary outcome measure was spasticity assessment per the gold standard clinical scale, the Modified Ashworth Scale. Secondary clinical outcome measures were the muscle force, pain, balance, trunk control, independence in walking as well as daily living independence, and patients’ experience, in terms of acceptability and usability. Robot forces during training were also considered as secondary instrumental outcomes. Research was conducted in an ethical and responsible manner, following the principles of the Declaration of Helsinki. The local ethical committee at Fondazione Santa Lucia IRCCS approved the study, and all patients provided written informed consent to participate (CE\AG4\PROG 329). Twelve consecutive stroke in-patients admitted to Fondazione Santa Lucia IRCCS were enrolled in the study according to the following inclusion criteria: age > 18 years, non-ambulatory patients, first-ever stroke, time elapsed since stroke occurrence from 3 weeks up to 6 months (subacute phase), and presence of an unilateral lesion. The exclusion criteria were: global cognitive deterioration, severe comprehension-impaired communication, drug treatment affecting awareness, other concomitant neurological disorders (e.g., Parkinson’s disease), severe concomitant diseases (metabolic disorders, severe cardiac impairment), severe symptomatic orthostatic hypotension, gross dystonic/involuntary movements, high level of spasticity (Modified Ashworth Scale higher than 3), pressure sore of stage 2 or higher, debilitating diseases that cause exercise intolerance, or severe reduction in the lower limb joints’ range of motion.

Patients’ epidemiological features are reported in Table 1. From the total cohort of 12 patients, 2 patients dropped out after enrolment, one due to pain onset at the affected upper limb (PT5) and one due to an episode of an epileptic crisis during conventional rehabilitation (PT6). These problems were not related to the robotic training.

**Table 1:**
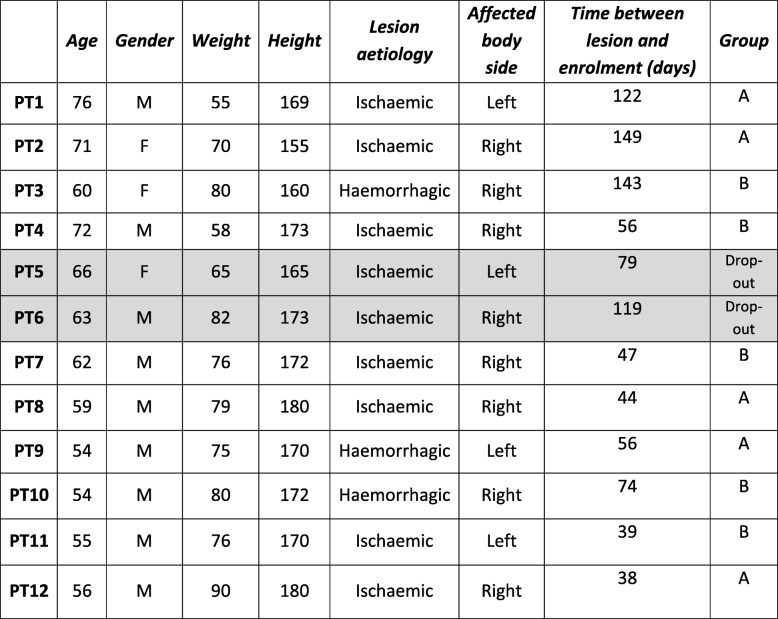
Patients (PT) epidemiological features. Patient allocation in Group A or B is also reported. Grey lines refer to patients who did not complete Lokomat training (PT5 and PT6)

### Intervention

**Fig. 1:**
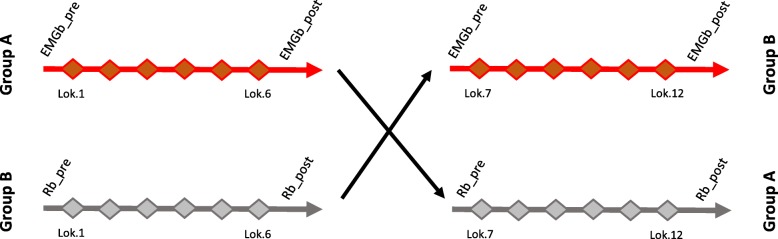
Randomized cross-over case control clinical trial schema. Group A stroke patients underwent 6 EMGb followed by 6Rb Lokomat trainings. Group B stroke patients underwent 6 Rb followed by 6 EMGb Lokomat trainings. For each subject the total amount of training was of 12 sessions

After enrolment, patients were randomized by a randomization electronic list into two treatment groups, A and B, each with 6 patients, based on the two different biofeedback conditions used during training. For both groups, the study protocol consisted of 12 sessions of Lokomat training, 6 for each biofeedback condition, with a duration of 40 min each, including donning and doffing the harness, with a frequency of 3 sessions per week. All patients performed Lokomat training as an add-on therapy to their conventional rehabilitation. The conventional rehabilitation protocol consisted of 40 min per day, 5 days per week, and was the same for both groups. So, the total amount of rehabilitation per patient was the same for both groups A and B. Group A (*N* = 6) underwent 6 EMGb sessions, followed by 6 Rb sessions. Group B (*N* = 6) underwent 6 Rb sessions, followed by 6 EMGb sessions (see Fig. 1).

Clinical, behavioural assessments and robot measurements were performed for both groups at enrolment and after the 6th and 12th training sessions.

Concerning the robotic training settings, for each patient and for each biofeedback session, the body weight support (BWS) was set at 50% of the body weight and maintained as constant during all 12 training sessions. Guidance assistance was maintained constant at 100%, and the gait speed was always 1.3 Km/h. During each session, the same physical therapist was always with the patient, providing guidance via verbal instructions on biofeedback management [33] .

### EMG-based biofeedback

**Fig. 2:**
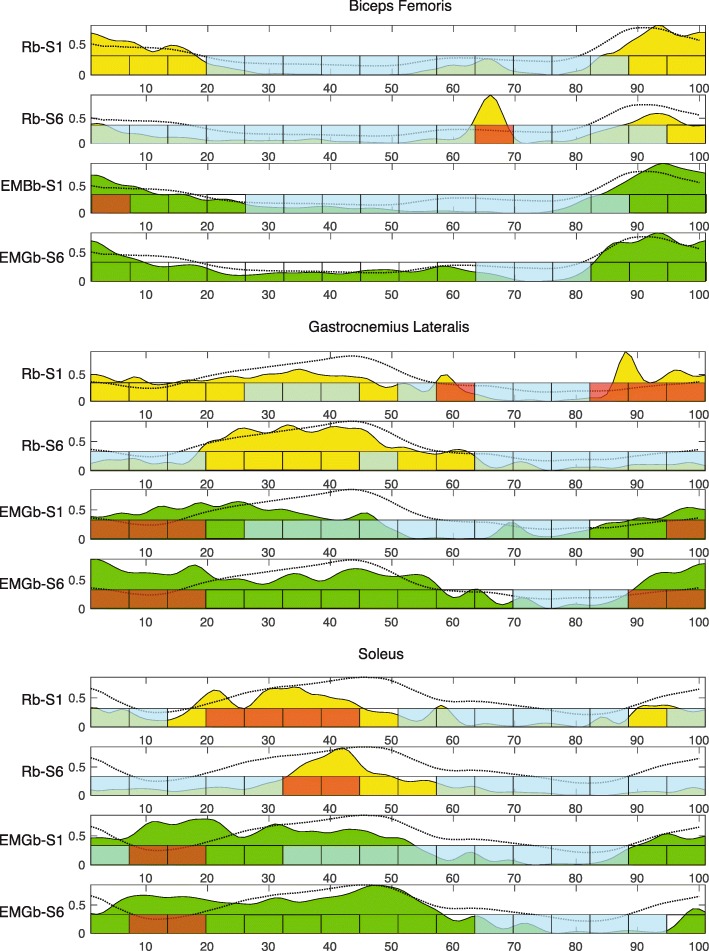
Representative average muscle activation of biceps femori, gastrocnemius and soleus for the first (S1) and the last (S6) training session with EMGb and Rb for PT1 (shadowed area in yellow for Rb and green for EMGB). Reference activation pattern used to compare against to compute the biofeedback (dotted curves); 16 blocks of biofeedback during the gait cycle with colour representing the assessment of muscle activation (red is underactivation with respect to the reference, blue is overactivation with respect to the reference, transparent is no deviation)

**Fig. 3:**
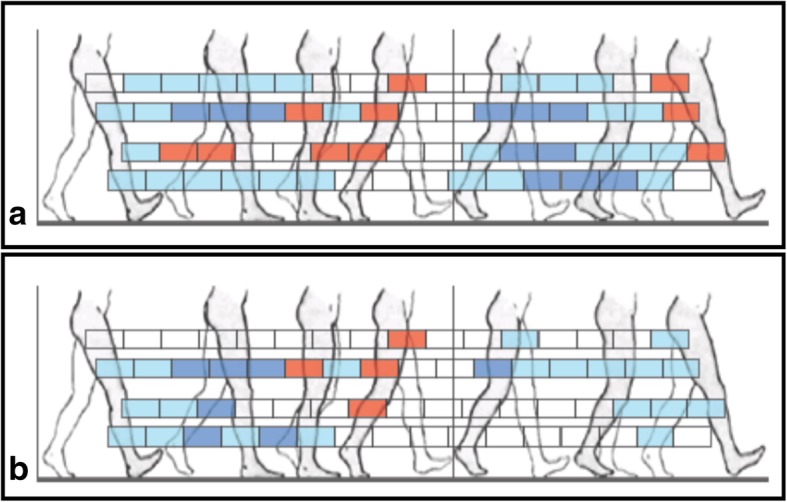
Representative image of visual biofeedback provided to the patient (PT6) according to on-line EMG activity during first (**a**) and last (**b**) EMGb training session. EMG data were displayed on the screen with 4 colour stripes partitioned into 16 stages within the gait cycle. First stripe referred to VL-RF, second stripe refers to BF, third stripe referred to GM-SOL and last stripe referred to TA. Coloured lines in the patient’s feedback were generated as follows: i) Red colour means that the signal is higher than in the template, or ii) Blue means that the signal is lower than in the template. From Fig. 3-b is evident a more physiological muscle activity during the whole gait cycle

Electromyographic data was acquired at 256 Hz with a notch filter at 50 Hz (g.USBamp biosignal amplifier, g.tec Austria) with sixteen active leads (g.GAMMAclip, g.tec medical engineering GmbH, Austria) attached to disposable Ag/AgCl electrodes that were placed in accordance with SENIAM guidelines [34] on the tibialis anterior (TA), gastrocnemious lateralis (GL), soleus (SOL), vastus lateralis (VL), rectus femoris (RF), biceps femoris (BF) of the affected leg. Connection wires were clipped on surface electrodes on one side and connected to the amplifier in a workstation. The workstation acquired electromyographic data and implemented functions to receive data from the Lokomat (kinematics, forces and digital trigger events, e.g., the beginning of the stance phase for left and right legs) and to transmit data for visual feedback. These functions are implemented in MATLAB/Simulink (The MathWorks Inc., Natick, MA) using a rapid prototyping environment (RPE, i.e., g.HIsys, g.tec medical engineering GmbH, Austria). In particular, the novel EMGb was implemented including an online intuitive graphical user interface (GUI) representing muscle activity. The developed biofeedback takes advantages of a GUI consisting of 2D silhouettes of the affected lower limb and providing on-line levels of activation of VL, RF, BF, TA, GL and SOL muscles of the patient’s affected leg. In particular, the EMGb was constructed to visually inform the patient about the comparative level of activation of each muscle with respect to a targeted reference muscle activation profile (Fig. 2). The targeted reference muscle activation profile provided was obtained from EMG signals acquired in a previous study [35], involving healthy volunteer subjects walking in the Lokomat set to 0% guidance force which means that the robot was following the movement of the subject without interfering. Average rectified values were computed to obtain the reference pattern. Then, an electromyographic -based biofeedback was implemented that focused on specific phases of the gait pattern in four muscle groups (VL-RF, BF, GM-SOL and TA). These data were displayed on the screen in 4 stripes partitioned into 16 stages within the gait cycle, each stage indicating over-activation (denoted in blue colour) or under-activation (denoted in red colour). The colouring of the stripes was based on the calculation of the muscle activation index, the floating point value of which ranged from − 1 (under-activation) to + 1 (over-activation), with a value of 0 indicating optimal muscle activation (denoted in white colour). In EMGb training, patients were requested to adapt their muscle activation of the four muscle groups according to the activation colour code visualized (Fig. 3). The algorithm sequence to extract the muscle activation for each gait cycle was the following: a) Data were acquired with a sample rate of 256 Hz and a notch filter at 50 Hz; b) Data triggering with the “step detection” signal provided by the Lokomat PRO system’s “Ouput Box”; c) Bandpass filtering (Butterworth 2nd order): high-pass 10 Hz; lowpass 100 Hz; d) Calculating absolute value of data; e) Bandpass filtering (Butterworth 2nd order): high-pass 0.0001 Hz; low-pass 10 Hz; f) Down sample data by factor 4; g) Normalization of each triggered segment with respect to its maximum value. Data were time normalized between zero and one for each gait cycle. The filtered and time normalized EMG values were averaged within each of the gait phases, downsampled to the number of values of the reference signal and then its absolute vale was compared to the reference dataset. The coloring of the stripes was updated based on calculation of the activation index, whose floating point value ranged from − 1 (under-activation) to + 1 (over-activation), with value equal zero indicating optimal muscle activation.; h) The triggered data were compared to the template file of the targeted muscle activation profile; i) Colouring lines in the patient’s feedback as follows: 1) Red colour means that the signal is higher than in the template, or 2) Blue means that the signal is lower than in the template; j) Colour lines are created with the “surface” function in Matlab.

### Joint torques-based biofeedback

**Fig. 4:**
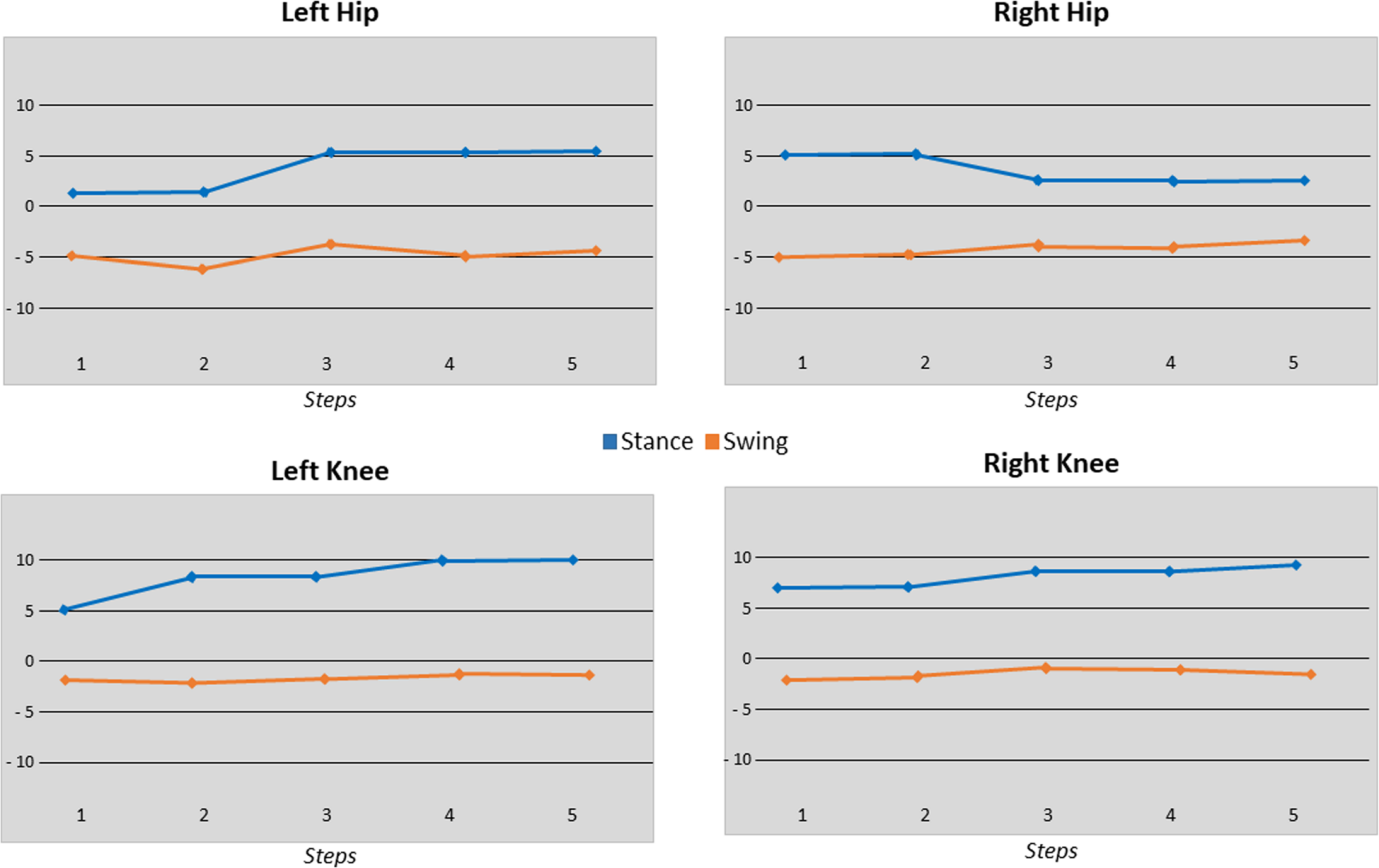
Standard display of commercial joint torque biofeedback (Rb) implemented in the Lokomat for gait training. BFB values are available for the right and left hip and knee joints as well as for stance and swing phases. Each point represents the BFB value of one stride. Data are displayed in a line diagram, which is updated for each stride and torque values are displayed in independent subplots for each one of the four joints. Swing and stance phase are color-coded. In this Figure a positive feedback is provided for all joints, especially for the knees, during stance phase indicating that the patient actively moves joints according to the reference trajectories, while during the swing phase, particularly for the hips, patient dos not contribute to the walking movement than the robot has to exert torque in order to maintain the desired reference trajectory

For the Rb, biofeedback values were calculated for the stance and swing phases of the gait cycle as weighted averages of the torques measured in the corresponding joint drives. Appropriate selection of the weight functions lead to positive biofeedback values when the patient performs hypothesized therapeutically desirable activities. Specifically, active hip flexion is required to bring the leg forward during the swing phase, active knee flexion during the early swing phase and knee extension during the late swing phase. During the stance phase, the most important activity was weight bearing by a continuous, almost isometric knee extension, whereas a hip extension results from a combination of muscle activity and passive motion of the treadmill [25]. The complete display, placed in front of the patients, showed all values per stride in an array of line graphs, each including the history for a number of five recent strides. No ankle information was displayed on the screen (Fig. 4).

### Data collection

From the total cohort of 12 patients, 10 (group A: *N* = 5, group B: *N* = 5) completed the entire protocol. For both Groups A and B, before (EMGb_pre and Rb _pre) and at the end of the six EMGb or Rb trainings (EMGb_post and Rb_post), a battery of clinical, neurological and psychological assessments as well as robot measurements were collected for each patient, as detailed below. All assessments were performed by the same operator, who was blinded to the type of biofeedback, at the same time interval from the last treatment session for both groups. The treatment effects due to the biofeedback were analysed by grouping the Rb and EMGb data of the group A and B patients.

### Clinical and neurological assessment

As concern clinical and neurological assessment, primary and secondary outcome were defined. Considering the main aim of the study and the different electromyographic contents tested, the Modified Ashworth Scale (MAS) was considered as primary outcome measure. Hip, knee and ankle spasticity for the affected limb were scored per the Modified Ashworth Scale (MAS), a point ordinal scale that grades resistance during passive stretching [36]. Secondary outcome measures were measured as follows. The Manual Muscle Test (MMT) [37] was used for a muscle force assessment of the hip, knee, and ankle muscles according to the motor strength grades of the Medical Research Council. Gait ability was addressed using Functional Ambulation Category (FAC) [38], with possible scores ranging from independent walking outside to non-functional walking, representing a patient who cannot walk or who needs help from 2 or more persons. Pain on the paretic side was assessed by using a Visual Analogue Scale (VAS) [39]. Also the Barthel Index (BI), the Trunk Control Test (TCT), and Berg Balance Scale (BBS) were addressed as secondary clinical outcomes.

The BI [40] was selected for the daily living independence assessment, and the BBS [41] and TCT [42] were used to classify balance impairments. The BBS can be considered a reflection of functional activity, and the TCT is a measurement scale that rates how well a patient is able to control trunk movements.

### Patients’ experience assessment: acceptability and usability evaluation

Besides daily living independence, balance and pain also patients’ experience were considered as secondary outcomes. Patients’ experience, in terms of acceptability and usability, was explored by means of patients’ mood, motivation and satisfaction assessments as well as their perceived workload. During the enrolment phase, patients were screened by means of the Center for Epidemiologic Studies Depression Scale [43], ranging from 0 to 60 points, with a cutoff of 16 points, above which individuals are considered to be at risk for clinical depression and were possibly excluded from the study. Motivation was assessed per the adapted version of the Questionnaire for Current Motivation (QCM), which was administered before starting each training session. The QCM is based on 4 motivational factors analysed by means of 18 statements: (1) “mastery confidence,” which refers to the certainty in succeeding at a task (4 statements); (2) “incompetence fear,” indicating the level of anxiety about failing in the task (5 statements); (3) “challenge,” denoting the perception of the task as a challenge (4 statements); and (4) “interest,” which indicates how much the task may or may not evoke interest (5 statements). Each factor is measured as the average score assigned to each statement belonging to that factor, ranging from 1 (“I completely disagree”) to 7 (“I completely agree”). A visual analogue scale (VAS) [39] was used for the assessment of motivation and mood factors before each training session, while a VAS for satisfaction evaluation was administered after each training session. The workload was measured by using the National Aeronautics and Space Administration Task Load Index (NASA-TLX) [44], considering the NASA-TLX to be an integrated measure of the overall workload. Workload is a hypothetical concept that represents the costs incurred by a human to achieve a particular level of performance. Workload consists of 6 component subscales: time pressure, own performance, physical effort, mental effort, frustration, and stress and fatigue. The NASA-TLX was administered at the end of the six training sessions of EMGb or Rb. After the 12th training session, patients were administered a modified version of the Quebec User Evaluation of Satisfaction with Assistive Technology 2.0 (QUEST 2.0) [45], a standardized satisfaction assessment tool for assistive technologies.

### Robotic measurements assessments

A detailed analysis of the impact of the treatments on the articular responses during the execution of the locomotor tasks on the robot was performed with primary biomechanical measures, namely, peak swing-phase hip and knee angular excursions and forces measured by the electromechanical drives of the robot. Again this data was considered as a secondary outcome. This particular assessment has been performed previously in a sub-group of patients with valid mechanical data. Due to the lack of complete data for some sessions in the entire group, only patients with available data for the first and last session of both EMGb and Rb were considered, ending with an analysis of a sub-group of patients (PT4, PT7, PT8, PT9, PT10).

### Statistical analysis

Descriptive statistics were assessed for all variables. Before statistical comparisons were made, a Kolmogorov-Smirnov test was performed to evaluate the distribution of the data. The treatment effects due to biofeedbacks were analysed by grouping the EMGb and Rb data of the group A and B patients.

A Wilcoxon test was used for the non-parametric clinical scales to compare the effects of the biofeedback approaches, evaluated as pre vs post data for each type of biofeedback (“EMGb: pre vs post”; “Rb: pre vs post”). Furthermore, the baseline data between EMGb and Rb (“Pre: EMGb vs Rb”) and the data after 6 days of Lokomat training between EMGb and Rb (“Post: EMGb vs Rb”) were compared.

Concerning robotic measures, from the measurements of the angle and forces of the hip (H) and knee (K) for every gait cycle of each session, the average force (F) values were calculated per patient for the affected and unaffected body sides. Measurements were divided for the swing and stance phases according to the minimum value of the hip angle. Using these data, we compared the first 20% gait cycles of the first session with the last 20% gait cycles of the last session with either EMGb or Rb, i.e., after 6 consecutive sessions using the same biofeedback. Statistical analysis was performed to compare these variables between EMGb and Rb by merging the data of the sub-group of patients. Likewise, statistical analysis was individually performed for each patient for each phase and each laterality. The analyses evaluated the hypothesis of the equivalence between the means of the variables with a confidence interval of 95%. The analyses were carried out by means of one-way ANOVA with Matlab software (MATLAB 2016b,© 1994–2018 The MathWorks, Inc.).

Furthermore, to more deeply understand the effects of possible improvements due to the biofeedbacks on patients’ experience, a Spearman correlation analysis was performed between usability and acceptability scales improvements versus the clinical and neurological performance data.

Statistical significance was considered at *p* < 0.05*.* All statistical tests were performed using the Statistical Package for the Social Sciences Software (SPSS), version 12.0 (Chicago, IL).

## Results

### Clinical and neurological assessment

No significant differences were present for the comparison between the EMGb and Rb groups at enrolment (EMGb_pre vs Rb_pre) for any of the clinical or instrumental variables analysed. Comparisons between groups at the end of either the EMGb or Rb training (EMGb_post vs Rb_post) did not show significant differences between groups for any of the assessments performed.

**Fig. 5:**
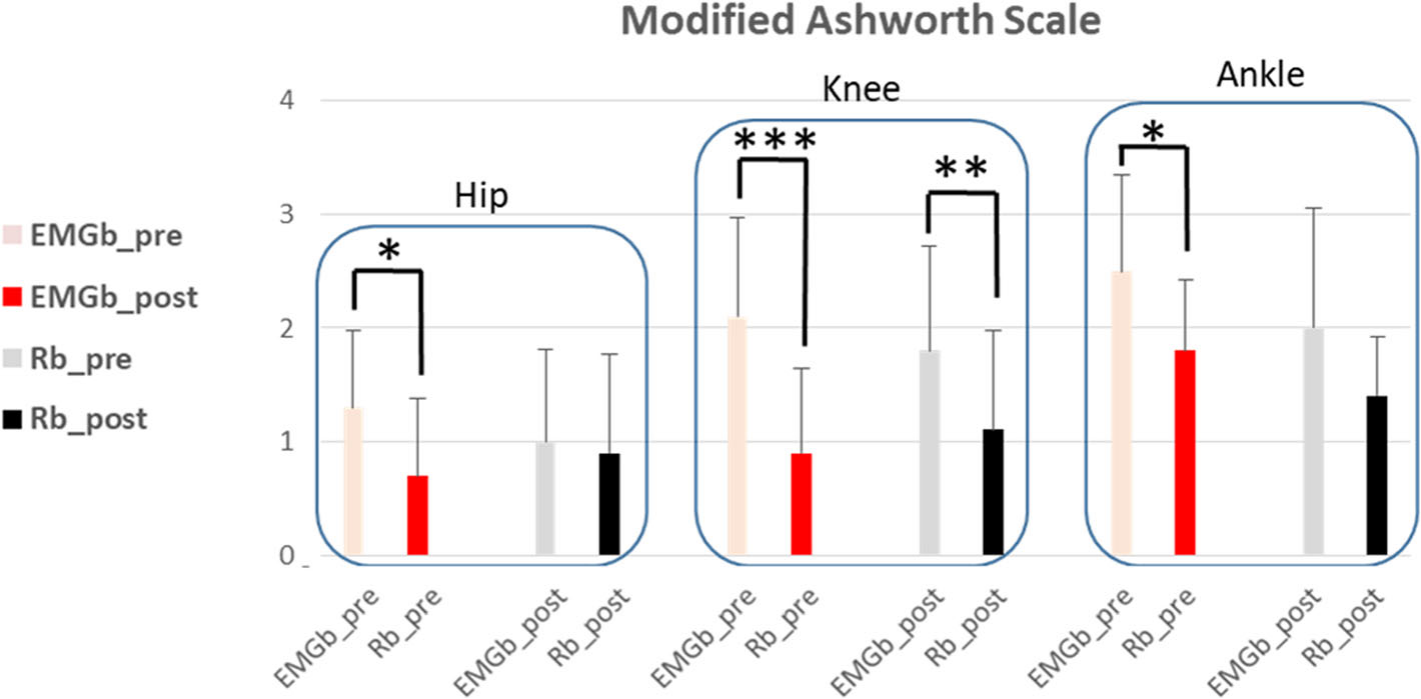
Modified Ashworth Scale (MAS) results at hip, knee and ankle, for the 10 patient’s cohort. Red columns refer to EMGb Lokomat trainings, while black one to Rb Lokomat trainings. For both EMGb and Rb groups, light columns represent MAS score before 6 Lokomat trainings (EMGb_pre or Rb_pre), while the darkest ones MAS score after 6 Lokomat trainings (EMGb_post or Rb_post). Statistical significance are reported for the comparison EMGb_pre vs EMGb_post and Rb_pre vs Rb_post (*: *p* < 0.05, **: *p* < 0.005, ***: *p* < 0.001)

**Fig. 6:**
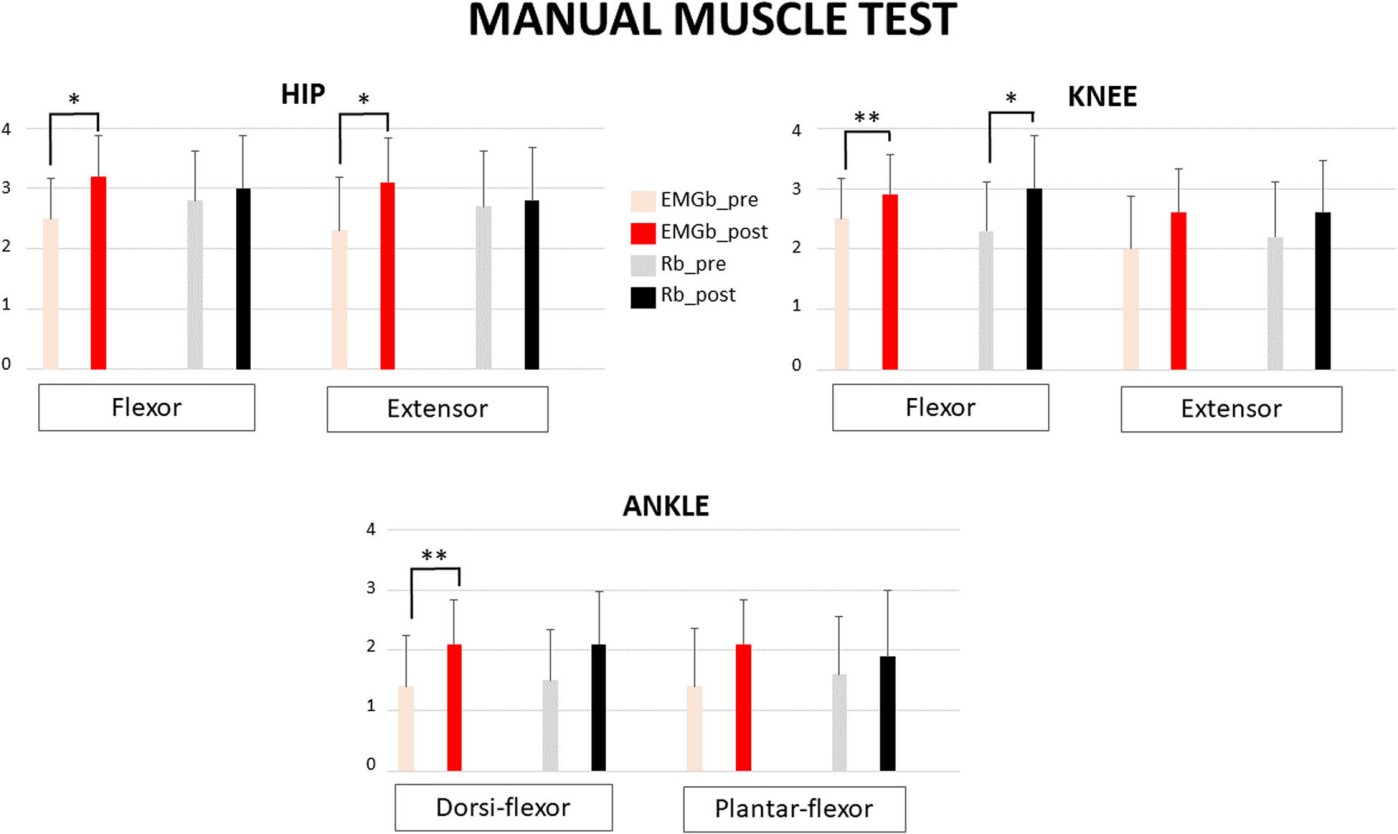
Manual Muscle Test (MMT) results for the 10 patients’ cohort at hip, knee and ankle flexor and extensor muscles. Red columns refer to EMGb Lokomat trainings, while black one to Rb Lokomat trainings. For both EMGb and Rb groups, light columns represent MMT score before 6 Lokomat trainings, while the darkest ones MMT score after 6 Lokomat trainings. Statistical significance are reported for the comparison EMGb_pre vs EMGb_post and Rb_pre vs Rb_post (*: *p* < 0.05, **: *p* < 0.005, ***: *p* < 0.001)

A comparison between the pre and post EMGb and Rb data demonstrated an improvement in all of the indexes considered. Regarding the primary outcome, MAS results after trainings demonstrated a significant spasticity reduction at the hip, knee and ankle for EMGb, while for Rb, only the knee spasticity reduction reached significance (see Fig. 5). The muscle force evaluation included all lower limb muscles, and muscle force improvements were generalized for all muscles in both biofeedback groups. Significant effects were limited in both groups to muscles working in the sagittal plane of movement, i.e., flexor or extensor muscles, although with interesting group differences (Table 2). In the EMGb group, significant improvements were present in the hip joint for both the extensor and flexor muscles, knee flexor and ankle dorsiflexor muscle force. Conversely, the Rb group significantly improved only in knee flexion force (Fig. 6). For the secondary outcomes, statistical significance was reached for both Rb and EMGb for FAC, Barthel index, and TCT, while for pain, a significant reduction in terms of VAS-score was only reached for EMGb. No statistical significance was reached for BBS in either the EMGb and Rb groups (Table 3).

**Table 2:** Manual Muscle Test results for the 10 patients’ cohort, EMGb and Rb Lokomat trainings groups as mean ± sd. Statistical comparison results are reported: Pre vs Post comparison for each BFBb and Rb groups. *p* values or “ns”, if statistical comparison is not significant, are reported for each statistical comparison performed

		EMGb		Rb		*Statistical Comparison*	
						*EMGb*	*Rb*
HIP joint	Hip-Flexion					*Pre* vs *Post*	*Pre* vs *Post*
		Pre	Post	Pre	Post		
	Mean	2,02	2,6	2,29	2,53	*0.035*	*ns*
	Sd	0,93	1,15	0,9	1,12		
	Hip-Extension						
	Mean	1,83	2,47	2,16	2,26	*0.011*	Ns
	Sd	0,83	1,14	0,85	0,96		
	Hip - Abduction						
	Mean	1,7	1,88	1,83	2,3	*ns*	*ns*
	Sd	0,93	0,84	0,83	0,96		
	Hip Adduction						
	Mean	1,72	1,98	1,84	2,16	*ns*	*ns*
	Sd	0,83	0,86	0,76	0,86		
KNEE joint	Knee Flexion						
	Mean	1,87	2,48	1,88	2,45	*0.004*	*0.04*
	Sd	1,02	1,27	0,76	0,97		
	Knee Extension						
	Mean	1,55	2,27	1,84	2,33	*ns*	*ns*
	Sd	0,95	1,15	0,76	0,72		
ANKLE joint	Ankle Flexion						
	Mean	1,18	1,92	1,38	1,84	*0.05*	ns
	Sd	0,67	0,85	0,95	0,87		
	Ankle Extension						
	Mean	1,3	1,83	1,47	1,84	*ns*	*ns*
	Sd	0,89	0,71	0,91	0,8

**Table 3:** Neurological and clinical assessment results, for the 10 patient’s cohort for EMGb and Rb Lokomat trainings groups as mean ± sd. In the last columns statistical comparison results are reported: Pre vs Post comparison for each BFBb and Rb group. *p* values or “ns”, if statistical comparison is not significant, are reported for each statistical comparison performed

	EMGb		Rb		*Statistical Comparison*	
	Barthel Index				*EMGb*	*Rb*
	Pre	Post	Pre	Post	*Pre* vs *Post*	*Pre* vs *Post*
Mean	27,43	41,04	30,36	42,88	*0.007*	*ns*
sd	16,97	15,37	11,88	11,68		
	Trunk Control test				*EMGb*	*Rb*
	Pre	Post	Pre	Post	*Pre* vs *Post*	*Pre* vs *Post*
Mean	32,07	47,48	33,65	44,6	*0.005*	*0.011*
sd	18,12	17,94	14,21	15,48		
	Functional Ambulation Category				*EMGb*	*Rb*
	Pre	Post	Pre	Post	*Pre* vs *Post*	*Pre* vs *Post*
Mean	0,74	1,66	0,85	1,41	*0.015*	*0.014*
sd	0,63	0,85	0,64	0,41		
	Berg Balance Scale				*EMGb*	*Rb*
	Pre	Post	Pre	Post	*Pre* vs *Post*	*Pre* vs *Post*
Mean	11,06	16,13	11,2	16,59	*ns*	*ns*
sd	4,59	6,24	3,31	3,54		
	Visual Analogue Scale				*EMGb*	*Rb*
	Pre	Post	Pre	Post	*Pre* vs *Post*	*Pre* vs *Post*
Mean	5,18	1,66	4,34	2,2	*0.011*	*ns*
sd	3,1	1,55	1,61	0,98		

### Patients’ experience assessment

**Fig. 7:**
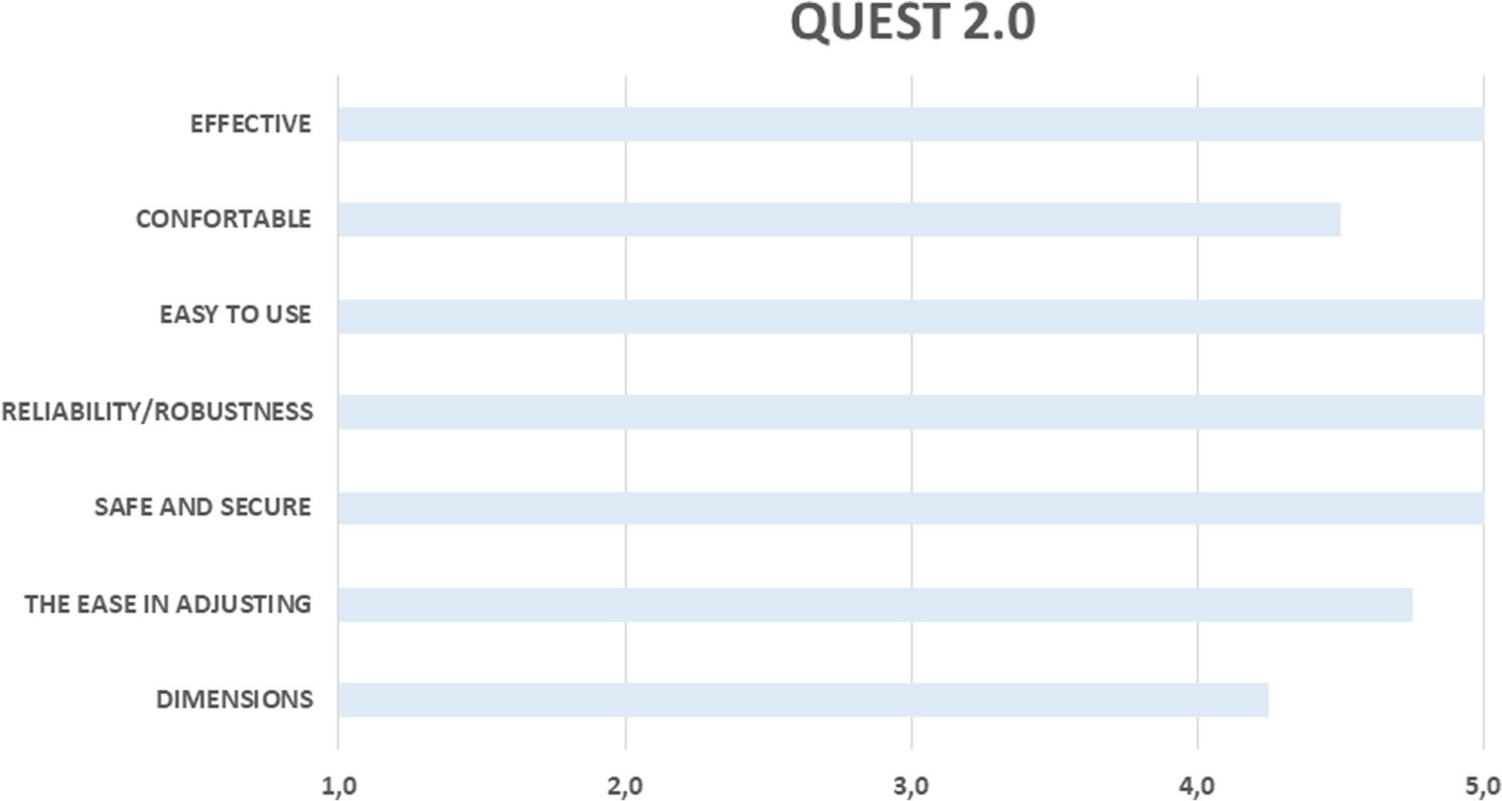
Acceptability and usability data of patients’ experience about Lokomat treatment per the QUEST 2.0 results

The patient sample was not at risk of depression as indicated by an average score of 5.76 ± 3.8 on the Center for Epidemiologic Studies Depression Scale. Regarding patients’ personal experience with the Lokomat, patients expressed a general positive attitude towards the robot. QUEST2.0 was used to assess the acceptability of assistive technology. QUEST 2.0 results showed a very high level of acceptability, and the robot was perceived to be highly effective, easy to use, reliable and safe (Fig. 7). It is worth noting that all patients were always assisted by an expert physiotherapist during training with the robot.

**Fig. 8:**
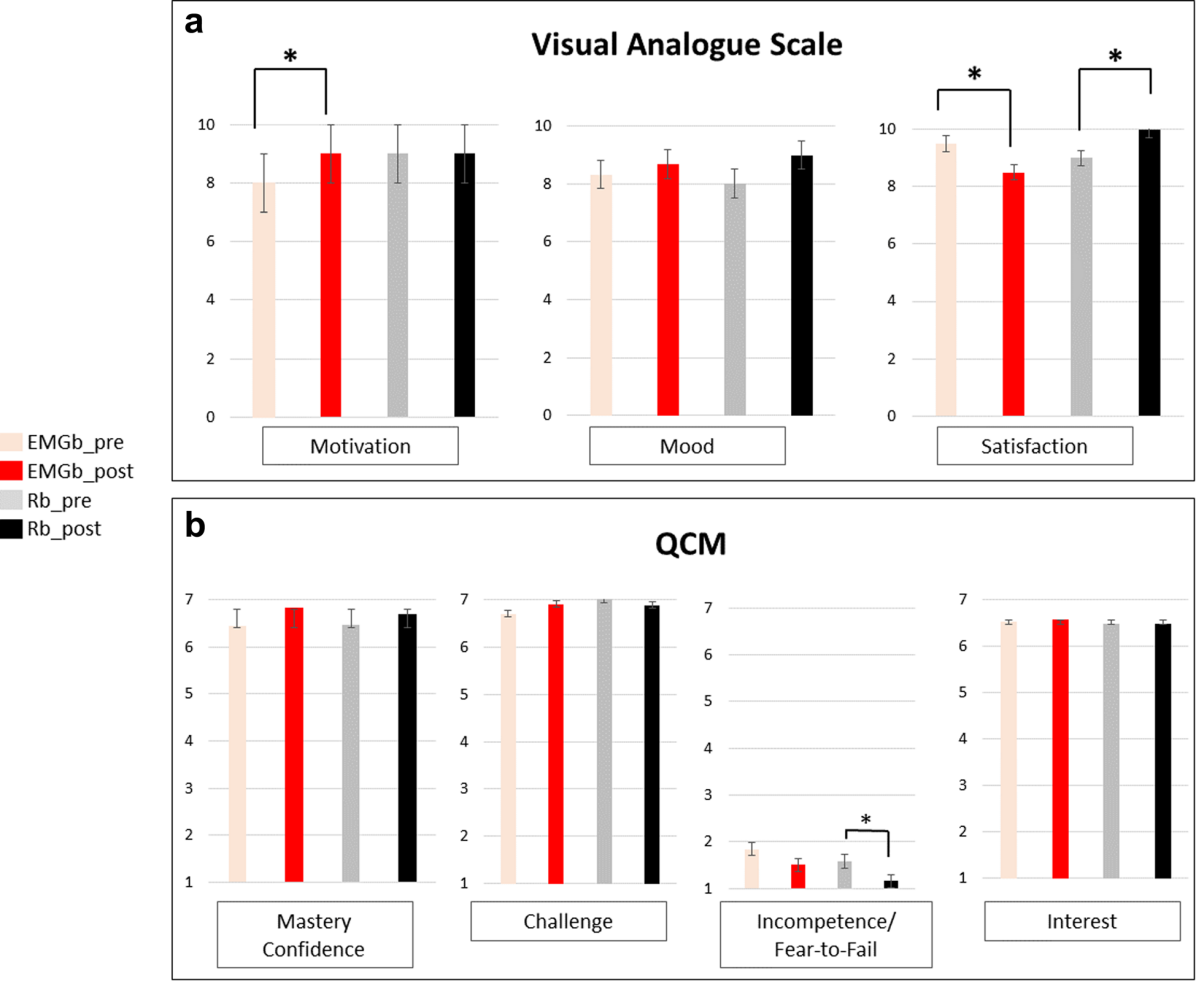
Mood, satisfaction and motivation data are detailed. Upper part of the figure (a) reports visual Analogue Scale (VAS) scales results about motivation, mood and satisfaction for the 10 patients’ cohort, while lower part of the figure (b) reports Questionnaire of current motivation (QCM) data for the 10 patients’ cohort. Red columns refer to EMGb Lokomat trainings, while black one to Rb Lokomat trainings. For both EMGb and Rb groups, light columns represent data score before 6 Lokomat trainings, while the darkest ones scores after 6 Lokomat trainings. Statistical significance are reported for the comparison EMGb_pre vs EMGb_post and Rb_pre vs Rb_post (*: *p* < 0.05, **: *p* < 0.005, ***: *p* < 0.001)

Furthermore, all patients rated their mood as “good” during the entire study, with some slight differences. The mood was rated as “very high” during sessions with both biofeedback conditions, with no statistically significant differences in either group in the comparison pre vs post. On the other hand, it was found that after EMGb Lokomat training, patients showed a significant increase in their evaluation of motivation, while satisfaction decreased. On the contrary, satisfaction significantly increased after Rb trainings, with no significant motivation changes (Fig. 8a).

In spite of the VAS motivation differences between groups, all of the QCM motivational sub-indicators factors were substantially stable. No significant differences were found between the pre vs post comparisons in either the EMGb or Rb trainings. For both biofeedbacks, patients showed a high degree of mastery confidence and a high level of interest throughout training, experiencing it as highly challenging. Surprisingly, no patient expressed any incompetence or fear-to-fail, even though it was the first ever robotic experience for all of them (Fig. 8).

Work load analysis, by means of the NASA-TLX questionnaire, revealed no significant differences in the perceived workload between the overall mean values obtained at the end of training: only a slightly lower rating for the Rb (total mean score = 42.86 ± 10.38), which was perceived as less demanding than the EMGb (total mean score = 48.13 ± 19.41).

### Robotic measures assessment

**Fig. 9:**
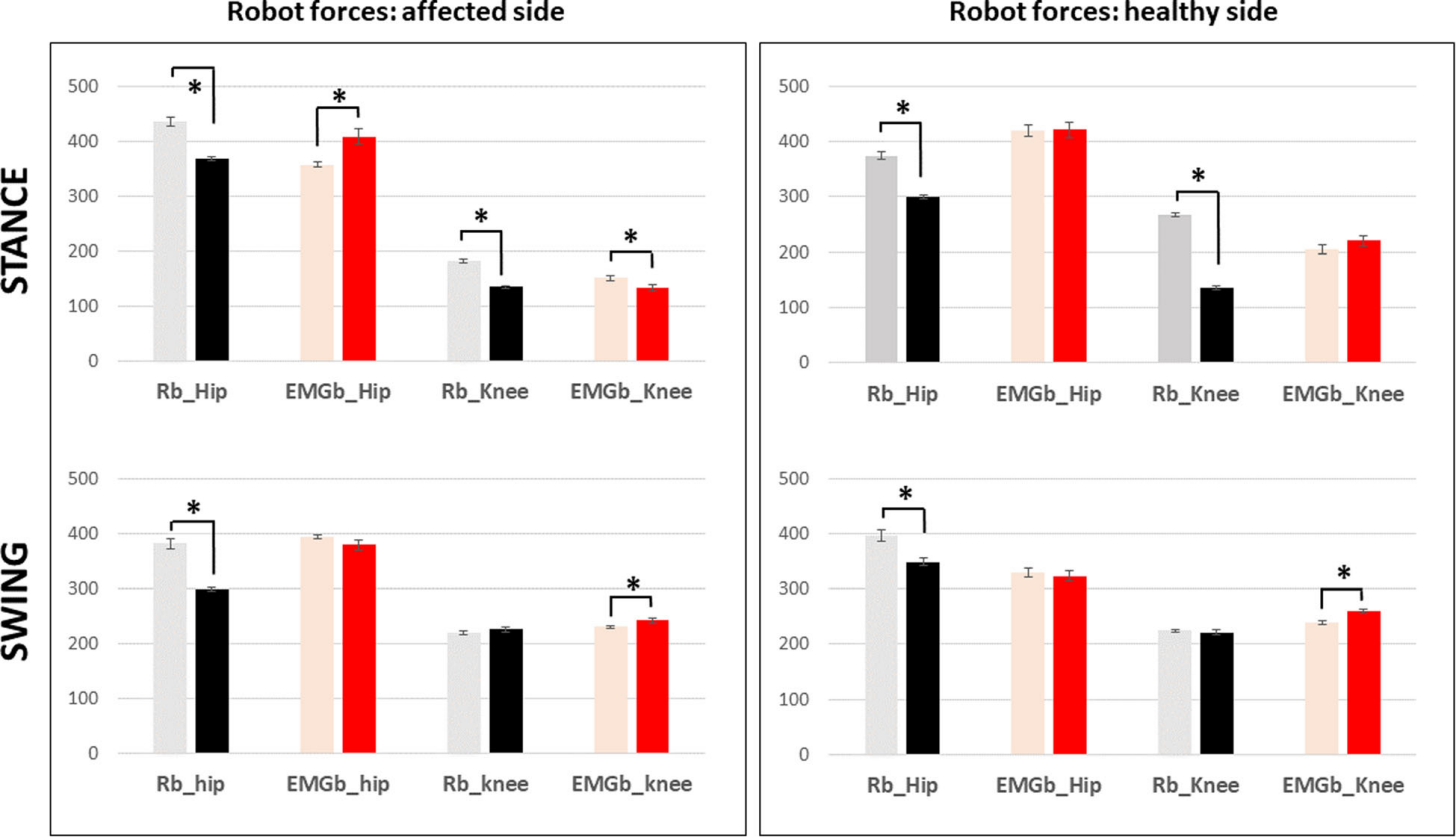
Mean joint forces of stance and swing phase for the affected and not affected leg in the subgroup of patients. Red columns refer to EMGb Lokomat trainings, while black one to Rb Lokomat trainings. For both EMGb and Rb groups, light columns represent the average score before 6 Lokomat trainings, while the darkest ones the score after 6 Lokomat trainings. Statistical significances are reported for the comparison EMGb_pre vs EMGb_post and Rb_pre vs Rb_post (*: *p* < 0.05, **: *p* < 0.005, ***: *p* < 0.001)

Robotic measurements of mean force data on the affected and healthy sides are reported in Fig. 9. Statistical comparisons between the EMGb and Rb groups, before and after the training sessions, failed to reach any significance. Within group analyses demonstrated pre versus post significant changes, with some differences between the two groups. In particular, for Rb after treatment, a significant reduction in the force exerted by the Lokomat was recorded for the affected and healthier legs in the hip during the swing phase and in the knee and hip during the stance phase. For EMGb after treatment, a significant increase in the force exerted by the Lokomat was observed in the knee during the swing phase and in the hip during the stance phase of the affected leg and only in the knee of the unaffected leg. A significant reduction of this force was present only during the stance phase in the knee of the affected leg. Overall, as depicted in Fig. 9, there was a tendency of the Rb to induce a more effective adaptation to robotic movements than observed after EMGb.

### Correlation analysis

Correlation analysis between the clinical results and patients’ experience, expressed as pre vs post data, was performed. Significant correlations were found only between the ankle MAS and VAS or QCM scores. Particularly for the EMGb group, the ankle MAS data negatively correlated with VAS motivation (*p* = 0.008): the ankle spasticity decrement was associated with an increase in daily motivation. On the contrary, during Rb training, the decreased level of spasticity of the ankle was positively correlated with the incompetence/fear-to-fail factor of the QCM (*p* = 0.007).

## Discussion

The present randomized cross-over clinical trial aimed to address the possible impact of different biofeedback contents on patients’ performance and experience during Lokomat RAGT, by comparing a novel biofeedback based on online biological electromyographic information versus the commercial joint torque biofeedback. Main differences between BFBs tested were: BFB content (EMG data vs joint torque data), the number of the joints for which the BFB was provided (hip, knee and ankle EMG data vs hip and knee joint torque data), the modality selected to represent BFB content (EMG data displayed as four groups in the GUI vs stance and swing torque data) and the timing (EMG data of a single step vs joint torque data of the last five strides). This study failed to demonstrate any significant differences in the effects of the different visual biofeedback-driven Lokomat gait trainings in non-ambulatory sub-acute stroke patients, according to the electromyographic and robot-based contents of the biofeedback. On the other hand, the results indicated biofeedback content specificity on the pattern of treatments’ effects as analysed by different clinical and instrumental assessments, particularly evident for the spasticity primary outcome.

There is a need for control-based studies on the effect of gait rehabilitation treatments. One of the main drawbacks is the lack of studies comparing two controlled therapies. In our study, we compared two treatments in which only one variable was changed, namely, the content of biofeedback information provided. As expected, in-line with previous studies on Lokomat RAGT [2, 13, 46], both groups presented a significant post treatment improvement of both clinical and performances indexes.

Lokomat RAGT is based on a task-specific repetitive rehabilitation approach [9], with high intensity [10] and early multisensory stimulation [11], for which motivation, active participation [15], learning skills [16] and error-driven-learning [17] are key aspects to improve patient robot interactions. Furthermore, Lokomat gait trainer provides a support to lower limb movements throughout the gait cycle along a pre-specified kinematic pattern that was obtained from normative gait data, known as robotic guidance force. In the Lokomat, an impedance controller allows to adapt the level of guidance force that acts as pulling force which brings the joint to the predefined path. Thus, in the robotic-guided walking condition provided by the Lokomat trainer in this study, the robot provided constant guidance force set at 100% at the knee and hip joints. We set this level of guidance to achieve normal-like gait patterns from the beginning of the longitudinal treatment even in non-ambulatory patients that had limited or were incapable of independent stepping. Ensuring a successful treadmill stepping pattern has shown to induce task-specific sensory information that could promote plastic changes in the central nervous system that are required to improve walking function after stroke [47]. In fact, it has been reported that promoting early task-specific robotic gait training with a top-down integration can improve gait recovery [48], even if previous published papers on Lokomat training effects are mainly based on a joint torque biofeedback and are mainly devoted to studying gait ability or independence in activity of daily living (ADL). Our group comparisons, highlight the specificities of the effects induced by treatment according to the biofeedback employed.

Considering that this is a novel study devoted to address possible Lokomat effects on lower limb spasticity in sub-acute stroke patients, particularly interesting are results regarding measures of spasticity, as well as muscle force data. In stroke population, spasticity can induce pain, tendon retraction or muscle weakness, which may limit the potential success of rehabilitation. Spasticity can also affect quality-of-life and be highly detrimental to daily function [32]. With regard to the MAS score, only for EMGb patients was spasticity significantly reduced for all lower limb joints, while Rb training only allowed a knee spasticity reduction. Parallel to the spasticity enhancements, an improvement in muscles’ strength was found for the hip, knee and ankle in the EMGb group, but only in the knee flexor for Rb group. The higher positive spasticity effects of the EMGb are in agreement with the findings of Tamburella et al. [33], who demonstrated the efficacy of a visual electromyographic-based biofeedback for ankle spasticity recovery in stroke patients. In the context of stroke rehabilitation, ankle recovery is considered a crucial goal for the subsequent recovery of ambulation [33], and it is plausible that the absence of ankle information during Rb may compromise the ideal expected effect of motor learning. Real-time wide-ranging biofeedback relative to all lower limb joints, as in the case of EMGb, is therefore a necessary training to maximize motor recovery, as suggested by Hidler [46]. Furthermore, to date in the literature, there are no studies aimed at evaluating the efficacy of training with Lokomat on affected limb pain perception in patients suffering from an ictal event, considering pain as a spasticity-related symptom [49]. Our treatment data proved there was a reduction in the VAS score with respect to the initial assessment for both groups, even if this improvement was statistically significant only for patients undergoing EMGb, suggesting that a biofeedback based on electromyographic information is more useful for managing muscle force, spasticity and pain spasticity-related symptoms with respect to a joint torque-based biofeedback.

Data on robot forces are interesting and indicate differences in effects according to the biofeedback used. Treatment with a joint torque biofeedback significantly reduced the Lokomat exerted forces for affected and not affected legs at the hip in the stance and swing phases and at the knee in the stance phase only. Conversely, treatment with EMGb significantly increased the Lokomat exerted force for the knee joint during the swing phase in both the affected and not affected legs and in the affected hip during the stance phase only. A force reduction was only present for the affected knee during the stance phase. Overall, Rb presented a more positive influence on patients’ compliance, allowing a more diffuse reduction of the forces exerted by Lokomat in respect to the EMGb.

In addition to performance, it is interesting to understand patients’ perception of the visual feedback content as a tool to influence treatment. Usability and acceptability tests are useful for generating direct data on user interactions with such technological tools and are thus essential for assessing their impact and acceptability in a rehabilitation setting. In this study, all patients had a good experience during robotic training with both types of tested visual biofeedback (EMGb or Rb), with a good mood and a high level of motivation maintained throughout the sessions. Particularly, EMGb significantly enhanced the level of mood. This interesting result, that Lokomat training creates a general positive attitude through the usage of the robot, goes beyond the biofeedback content comparison, even if a deeper investigation of patients’ experience suggests some differences in users’ perception. Personal involvement of patients during Lokomat training was calculated by correlating clinical scales data and usability and acceptability scores. The results show that a clinically evidenced ankle spasticity reduction allows for an increase in the daily motivation for EMGb training, while increasing the perception of incompetence and fear-to-fail for Rb training. It is worth noting that the amount of conventional rehabilitation and physical therapist assistance were the same for both biofeedback conditions during training, and the only difference was the type of biofeedback used. Thus, correlation differences between the two biofeedback types are intriguing but not easy to explain. We hypothesize that visual information provided directly to the patients on the ankle muscles in the case of EMGb may help them to better cope with ankle spasticity because of the online biofeedback. It is possible that EMGb can stimulate patients to constantly improve their performance. Conversely, in the case of Rb training, patients are unaware of their ankle muscle activity, as well as the robot forces, generating a higher level of incompetence and fear-to-fail due to the unawareness of performance. This is in line with previous studies showing how robotic walking training should be tailored on patients’ clinical and even psychological features [50].

In this study we enrolled stroke subjects with a mean age of 62,33 ± 7.49 years. The incidence of stroke rapidly increases with age, doubling for each decade after age 55 [51] and over 70% of all strokes occur more or less at the age of 65 years [52]. Furthermore, residual disability associated with stroke, in addition to presence of other chronic illnesses at the time of the stroke, makes stroke one of the most feared consequences of aging [52]. Only 5/10% of acute cerebrovascular events occur in people younger than 45 years of age. In this sub-population of young adults, the motor outcome of cerebral damage is better than in older patients [53]. Thus, we can speculate that BFB effects obtained in old stroke adults, may be more evident in younger stroke population.

### Limitations of the study

Despite our encouraging results, we conducted this cross-over pilot study with a small number of patients (*N* = 10). However, statistical significance obtained on a small sample of patients could indicate an even greater significance if the study were extended to a larger number of patients [54], also in stroke population [33]. Furthermore, the data collected may allow for the calculation of the sample sizes needed to achieve statistical significance in future studies, studies that also need to investigate longer treatments effects or possible follow-up assessments.

## Conclusion

High-quality evidence can be generated by performing a randomized controlled trial when evaluating the effects of an intervention [31]. In this study for the first time we directly compared EMGb versus a joint torque-based biofeedback, Rb, during Lokomat gait training in a randomized cross-over clinical trial in non-ambulatory stroke patients. Comparisons between the electromyographic and robot-based data suggest the importance of the biofeedback content during RAGT.

Overall comparisons between the two sets of data indicate that functional specific effects can be related to the biofeedback content: mainly when muscular-based (electromyographic data) biofeedback information is used, a more direct effect on muscle activity is evidenced for all lower limb joints. In a similar manner, when joint torque data are used to feed the biofeedback protocols, then a more diffuse effect on patients’ compliance with the robot movements is achieved. This latter aspect is clearly indicated by the observed post treatment reduction of the Lokomat exerted forces after Rb than after EMGb. Considering the differences between BFB contents tested, and also the differences related to the number of joints for which the BFB was provided, the modality selected to represent BFB content and the timing of BFB information, further studies devoted to better clarify the influence of these single BFB components on subjects performances should be done.

## Data Availability

Data and code are available upon request.

## Abbreviations

ADL: Activity of Daily Living
AS: Visual Analogue Scale
BF: Biceps femoris muscle
BI: Barthel Index
BWSS: Body weight support systems
EMG: Electromyographic
EMGb: Electromyographic-based biofeedback
FAC: Functional Ambulation Category
GL: Gastrocnemii lateralis muscle
MAS: Modified Ashworth Scale
MMT: Manual Muscle Test
NASA-TLX: National Aeronautics and Space Administration Task Load Index
PT: Patient
QCM: Questionnaire for Current Motivation
QUEST: Quebec User Evaluation of Satisfaction with Assistive Technology 2.0
RAGT: Robot assisted gait training
Rb: Robot generated joint torque biofeedback
RF: Rectus Femoris muscle
SCI: Spinal Cord Injury
SOL: Soleus muscle
TA: Tibialis Anterior muscle
TCT: Trunk Control Test
VL: Vastus lateralis muscle
